# Pharyngo-jugular fistula after “salvage” total laryngectomy: a case report

**DOI:** 10.1186/s13256-015-0710-9

**Published:** 2015-10-06

**Authors:** Erika Crosetti, Andrea Fulcheri, Giovanni Succo

**Affiliations:** 1 ENT Department, Martini Hospital, Via Tofane 71, 10141 Turin, Italy; 2 ENT Department, S. Luigi Gonzaga Hospital, University of Turin, Regione Gonzole, 10, 10043 Orbassano, Italy

**Keywords:** Pharyngo-jugular fistula, Fistula, Complication after total laryngectomy

## Abstract

**Introduction:**

We present a rare case of pharyngo-jugular fistula in a patient who underwent salvage total laryngectomy after organ-sparing radiochemotherapy.

**Case presentation:**

A 77-year-old Caucasian man underwent total laryngectomy and bilateral neck dissection as salvage surgery after the failure of radiochemotherapy at another hospital. Thirty-five days after surgery, he was admitted to our emergency room for fever and massive oral bleeding during meals. Videopanendoscopy showed the presence of a large clot at the base of his tongue, while a neck computed tomography scan showed a pharyngo-jugular fistula with the presence of air in the left internal jugular vein. Cervicotomy was performed: the internal jugular vein was ligated and sectioned, and the pharyngeal defect was repaired with a pectoralis major myocutaneous flap. The postoperative period was uneventful. Twenty-five days post surgery, videofluorography showed the fistula had disappeared. Our patient then began oral feeding without complications and was discharged. At present, 5 years after the operation, our patient is alive and shows no evidence of disease.

**Conclusions:**

Pharyngo-jugular fistula is an uncommon complication after total laryngectomy, especially in the chemoradiation era, which is potentially fatal if not promptly treated.

## Introduction

Diagnosis and correct management of postoperative complications after head and neck oncological surgery is of substantial interest given the complexity of potential clinical conditions and associated morbidity, with an increase in the duration of hospitalization and related costs. The increasingly widespread use of organ-sparing radiochemotherapy protocols has led to the use of salvage surgery only in cases of persistent or recurrent disease [1], which is, however, associated with a greater incidence of major complications compared to planned posttreatment neck dissection (24–61%) [1–3]. In salvage total laryngectomy, the most frequent early complications are pharyngocutaneous fistula, wound dehiscence and postoperative hemorrhage; common late complications include dysphagia and stenosis of the tracheostomy [4].

In this case report, we present a rare case of pharyngo-jugular fistula in a patient who underwent salvage total laryngectomy after organ-sparing radiochemotherapy.

## Case presentation

A 77-year-old Caucasian man was admitted to our department, complaining of two episodes of massive oral hemorrhage during meals, accompanied by odynophagia and fever. Thirty-five days before, in another hospital, our patient had undergone total laryngectomy and bilateral selective neck dissection after failure of organ-sparing salvage radiochemotherapy (cisplatin 100mg/m^2^ every 21 days for three cycles, and standard radiotherapy: 70Gy, 1.8Gy/day). The postoperative period was complicated by a small pharyngocutaneous fistula, which resolved in a few days with compressive bandages.

**Fig. 1:**
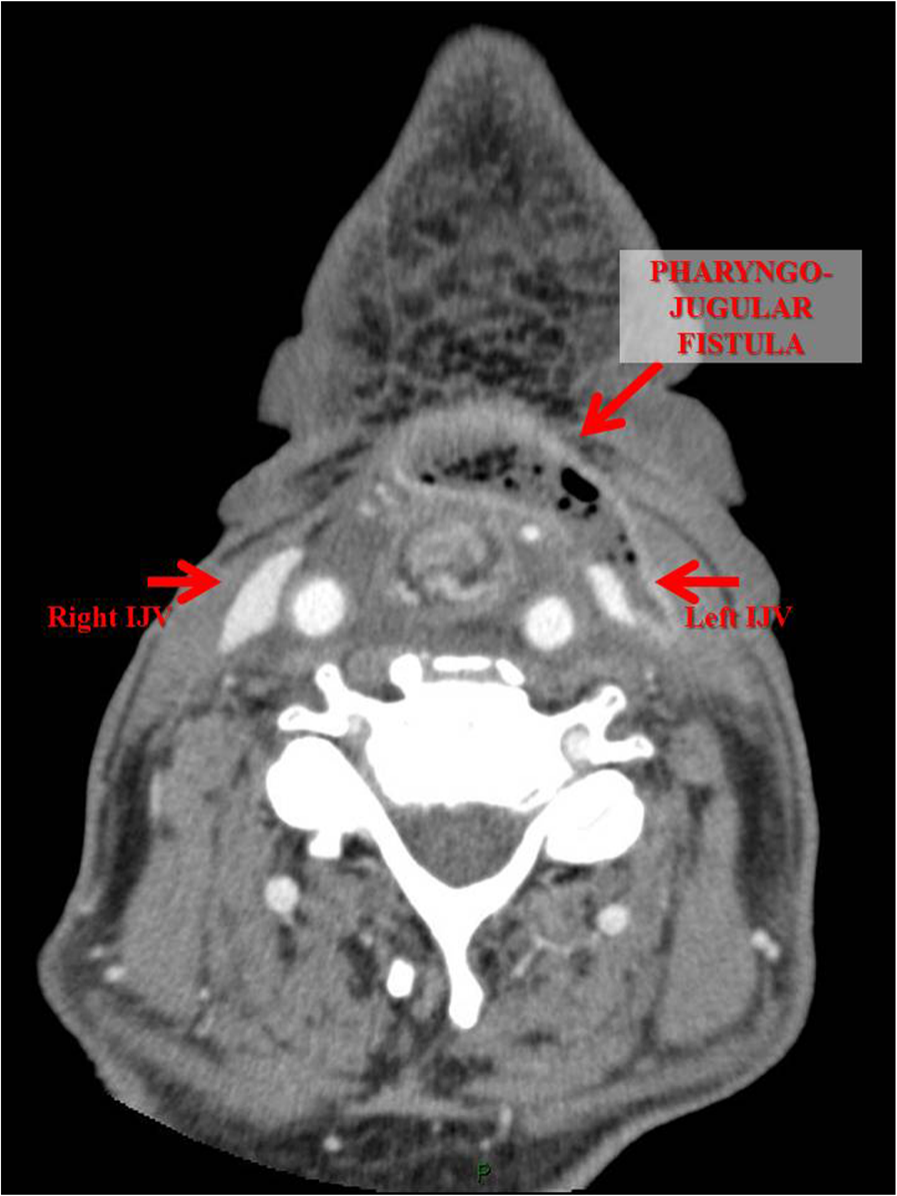
Neck computed tomography (axial view) showed the presence of a fistula between the left internal jugular vein and pharynx

**Fig. 2:**
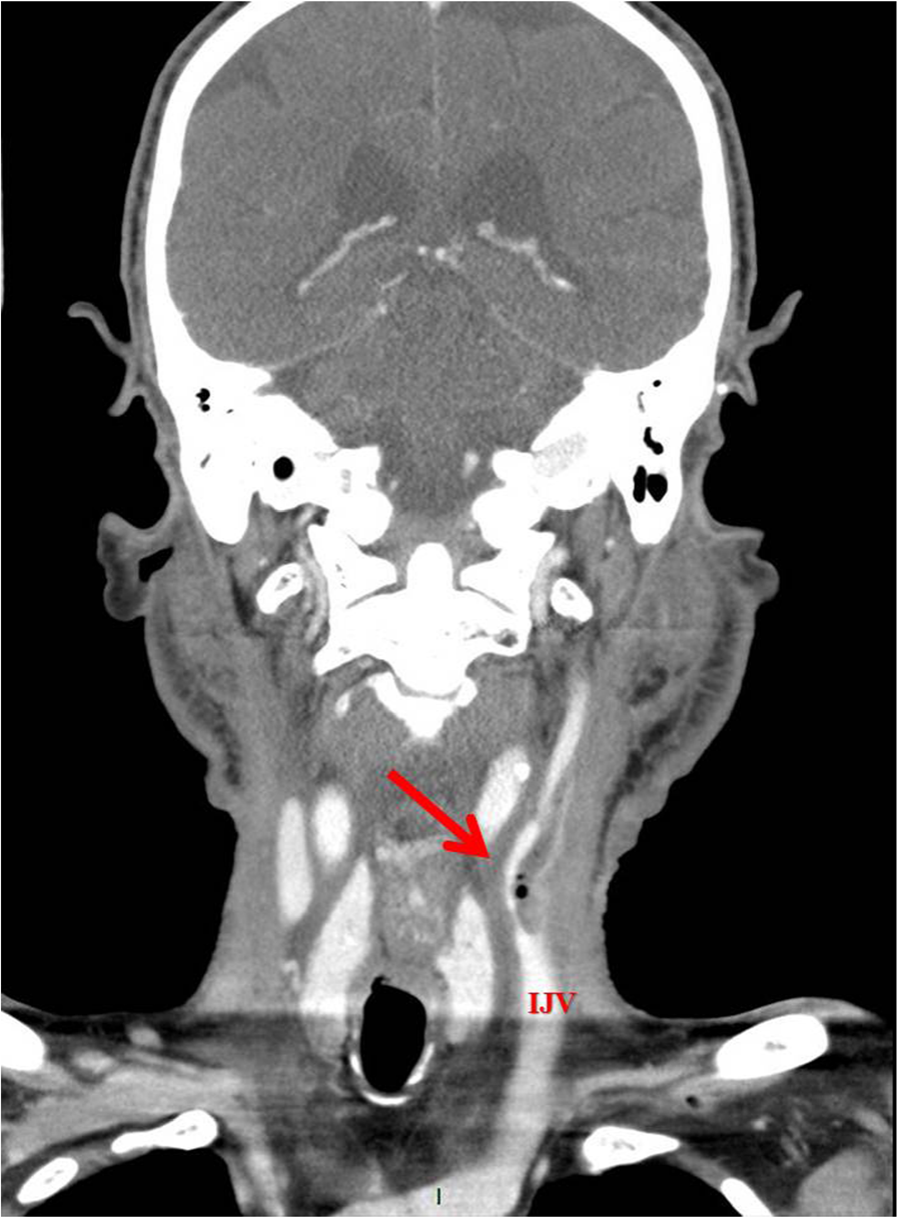
Neck computed tomography (coronal view) revealed a fistula between the left internal jugular vein and pharynx

Videoendoscopy demonstrated the presence of a large clot at the base of his tongue. A neck computed tomography (CT) scan (Figs. 1 and 2) revealed a pharyngo-jugular fistula with the presence of air in the left internal jugular vein (IJV). Our patient was subjected to an emergency operation consisting in cervicotomy, ligation and section of the left IJV and closure of the fistula using a pectoralis major myocutaneous flap.

The postoperative period was uneventful. Twenty-five days post surgery, videofluorography was performed, which showed the fistula had disappeared. Our patient began oral feeding and was discharged. At present, 5 years after the operation, our patient is alive and shows no evidence of disease.

## Discussion

Salvage surgery of the neck and upper aerodigestive tract in the era of chemoradiation is unfortunately associated with a greater incidence of major postoperative complications compared to primary surgery, with an increase in morbidity, mortality, duration of hospitalization and related costs [4–8]. In a recent article, in 24 patients previously treated with chemoradiation and subjected to total laryngectomy combined with total or partial pharyngectomy, Sewnaik *et al*. [3] reported a postoperative complication rate of 92%. Among early complications (within 3 months of operation), the most common were pharyngocutaneous fistula (50%), wound dehiscence (29%) and postoperative hemorrhage (21%), while among late complications dysphagia (25%) and stenosis of the tracheostoma were most frequent (21%).

In the literature, the reported rate of pharyngocutaneous fistula following total laryngectomy is around 16% after primary surgery [9], which increases to 19–50% after salvage laryngectomy [3, 10, 11]. Several authors have investigated the risk factors for pharyngocutaneous fistula after salvage laryngectomy, which include advanced stage of primary tumor [11], infection caused by methicillin-resistant *Staphylococcus aureus* [12] and previous radiotherapy [11].

Lee *et al*. [13] evaluated the risk factors for surgical site infection in patients undergoing major head and neck oncologic surgery. Infections at the site of operation, defined as infection of the surgical wound within 30 days following surgery and manifested as a purulent exudate or orocutaneous/pharyngocutaneous fistula, were observed in 10–45% of patients. Among the risk factors identified, there were both patient-related (co-morbidity, body mass index [BMI], tobacco use, alcoholism, American Society of Anesthesiologists [ASA] grade) and disease-related factors (site and stage of tumor), in addition to iatrogenic factors, such as previous radiochemotherapy, presence of tracheostoma, clean-contaminated wounds and extent of neck dissection [4, 6–8].

Morgan *et al*. [14], in a retrospective analysis of 38 patients subjected to salvage surgery after failure of organ-sparing radiochemotherapy for advanced head and neck tumors, reported the presence of pharyngocutaneous fistula in 20% of cases in whom pharyngotomy with successive pharyngeal suture was performed, and a single case of chylous fistula with carotid artery exposure in a patient subjected to planned neck dissection. As can be reasonably expected, the complication rate was lower in the group of patients undergoing neck dissection only compared to those subjected to neck dissection plus intervention at the primary tumor site, which also differed according to the type of wound complication (4% in the case of clean wounds, 23% for clean-contaminated).

Recently, Paydarfar *et al*. [15] carried out a systematic literature review on the risk factors for pharyngocutaneous fistula, which confirmed that the relative risk (RR) of developing a fistula after salvage laryngectomy was significantly higher after radiotherapy (RR 2.28 [1.59–3.25]) or radiotherapy associated with neck dissection (RR 2.96 [1.51–5.80]). Other risk factors included a reduced level of postoperative hemoglobin (<12.5g/dL, RR 2.10 [1.24–3.55]) and previous tracheostomy (RR 1.60 [1.05–2.44]). The severity and mean time of healing of a pharyngocutaneous fistula have been found to be significantly greater in patients with prior radiochemotherapy compared to those without preoperative radiochemotherapy [15].

To the best of our knowledge, this is the first report of a pharyngo-jugular fistula. In the present case, the postoperative period after the primary operation (total laryngectomy and bilateral selective neck dissection) was complicated by a pharyngocutaneous fistula that resolved with compressive bandages. It is possible that the bandage led to deviation and successive progression of the fistula toward the lateral cervical region, favoring pharyngocutaneous healing but with the formation of a pharyngo-jugular fistula.

In 2003, Cleland-Zamudio, in describing six cases of rupture of the IJV, observed a temporal relationship between rupture of the vessel and pharyngocutaneous fistula: the formation of the fistula generally preceded rupture of the vein [9].

In our opinion, a pharyngocutaneous fistula following salvage laryngectomy due to failure of an organ-sparing protocol is a common complication that can be resolved with either local dressing or reconstructive surgery. After apparent resolution of the fistula, it is important to perform videofluorography until it is completely resolved; the examination should always be carried out prior to reinitiating oral feeding. The use of pedicled or free flaps for reconstruction or the simple coverage of cervical vessels are valid surgical options in the attempt to reduce postoperative complications such as pharyngocutaneous and pharyngo-jugular fistula, with obvious benefits in terms of duration of hospitalization and associated costs.

## Conclusions

The increasingly widespread use of organ-sparing radiochemotherapy protocols has led to an increase in salvage surgical procedures in patients with persistent or recurrent disease [1], which are associated with a higher rate of complications compared to primary surgery [2, 3, 5–7]. Among these, pharyngocutaneous fistula is the most common.

The case described herein of a nonfatal pharyngo-jugular fistula is, to the best of our knowledge, the first such case described in the literature: this is a very rare complication after total laryngectomy, especially after salvage surgery, and is potentially fatal if not treated promptly.

## Consent

Written informed consent was obtained from the patient for publication of this manuscript and any accompanying images. A copy of the written consent is available for review by the Editor-in-Chief of this journal.

## Abbreviations

BMI: Body mass index
CT: Computed tomography
IVG: Internal jugular vein
RR: Relative risk
